# Unprecedented Mass Bleaching and Loss of Coral across 12° of Latitude in Western Australia in 2010–11

**DOI:** 10.1371/journal.pone.0051807

**Published:** 2012-12-17

**Authors:** James A. Y. Moore, Lynda M. Bellchambers, Martial R. Depczynski, Richard D. Evans, Scott N. Evans, Stuart N. Field, Kim J. Friedman, James P. Gilmour, Thomas H. Holmes, Rachael Middlebrook, Ben T. Radford, Tyrone Ridgway, George Shedrawi, Heather Taylor, Damian P. Thomson, Shaun K. Wilson

**Affiliations:** 1 Marine Science Program, Department of Environment and Conservation, Kensington, Western Australia, Australia; 2 Biodiversity and Biosecurity Branch, Department of Fisheries WA, Hillarys, Western Australia, Australia; 3 Australian Institute of Marine Science, Crawley, Western Australia, Australia; 4 The Oceans Institute, University of Western Australia, Crawley, Western Australia, Australia; 5 Pilbara Regional Services, Department of Environment and Conservation, Karratha, Western Australia, Australia; 6 Pilbara Regional Services, Department of Environment and Conservation, Exmouth, Western Australia, Australia; 7 Marine and Atmospheric Research, CSIRO, Floreat, Western Australia, Australia; University of Vigo, Spain

## Abstract

**Background:**

Globally, coral bleaching has been responsible for a significant decline in both coral cover and diversity over the past two decades. During the summer of 2010–11, anomalous large-scale ocean warming induced unprecedented levels of coral bleaching accompanied by substantial storminess across more than 12° of latitude and 1200 kilometers of coastline in Western Australia (WA).

**Methodology/Principal Findings:**

Extreme La-Niña conditions caused extensive warming of waters and drove considerable storminess and cyclonic activity across WA from October 2010 to May 2011. Satellite-derived sea surface temperature measurements recorded anomalies of up to 5°C above long-term averages. Benthic surveys quantified the extent of bleaching at 10 locations across four regions from tropical to temperate waters. Bleaching was recorded in all locations across regions and ranged between 17% (±5.5) in the temperate Perth region, to 95% (±3.5) in the Exmouth Gulf of the tropical Ningaloo region. Coincident with high levels of bleaching, three cyclones passed in close proximity to study locations around the time of peak temperatures. Follow-up surveys revealed spatial heterogeneity in coral cover change with four of ten locations recording significant loss of coral cover. Relative decreases ranged between 22%–83.9% of total coral cover, with the greatest losses in the Exmouth Gulf.

**Conclusions/Significance:**

The anomalous thermal stress of 2010–11 induced mass bleaching of corals along central and southern WA coral reefs. Significant coral bleaching was observed at multiple locations across the tropical-temperate divide spanning more than 1200 km of coastline. Resultant spatially patchy loss of coral cover under widespread and high levels of bleaching and cyclonic activity, suggests a degree of resilience for WA coral communities. However, the spatial extent of bleaching casts some doubt over hypotheses suggesting that future impacts to coral reefs under forecast warming regimes may in part be mitigated by southern thermal refugia.

## Introduction

Coral bleaching associated with ocean-warming events have received much recent attention and are responsible for a significant global decline in both coral cover and diversity over the past two decades [1], [2]. In particular, there has been an increase in coral bleaching over large spatial scales (100 s–1000 s kms) [3]–[8]. These ‘mass bleaching’ events are driven by ocean water temperature anomalies due to changes in global ocean-atmosphere circulation patterns, with the El-Niño Southern Oscillation (ENSO) cycle exerting the most influence over Australian waters [9]. The most recent mass bleaching events to severely impact reefs globally were in 1997–98 and 2005 [10], from which many are still recovering [11]. With the frequency of large-scale coral bleaching events expected to increase and become more severe, the consequences are likely to lead to the loss of critical services delivered by coral reef ecosystems [12]–[14].

Accurate assessments of threats to coral reefs are difficult to gauge at regional scales (100 s–1000 s kms) through the use of *in situ* methods. Remote sensing techniques using satellite-derived sea surface temperature (SST) observations are therefore used as an early warning system for predicting and monitoring mass bleaching events [15]–[17]. Cumulative thermal stress has been shown to be the best and most reliable predictor of coral bleaching [18]–[21], and is measured as the deviation above climatologically derived thresholds accumulated during a 12-week window [16], [22]. As indirect and direct effects of solar radiation can also influence bleaching extent and intensity [23], satellite solar irradiance products have also been derived to enhance predictive ability of remotely sensed products to monitor thresholds for coral bleaching [24].

The impacts of mass coral bleaching are varied and often difficult to assess, with regional scale patterns mediated through localised variability in physical and biological parameters [20], [25]–[27]. Notably, atmospheric conditions responsible for mass bleaching can also cause storms and cyclones [28], [29] confounding the effects of bleaching due to synergistic disturbance impacts. Additionally these physical impacts and resultant stress to corals also increases the incidences of disease [30], [31] and predation [32], [33].

Large-scale disturbances have previously had limited impact on the coral reefs of WA [1]. During the most severe global bleaching episode in 1998, there was localised bleaching at isolated offshore oceanic and atoll reefs off the continental shelf of WA [34], [35] with anecdotal reports of coral bleaching at other locations [4]. However, to date, there has been no documentation of widespread coral bleaching along continental reefs and adjacent islands of WA. During the 2010–2011 Austral summer, extreme La-Niña conditions resulted in record high and unprecedented regional-scale ocean temperature anomalies [36] and associated storminess and cyclonic activity. SSTs rose above long-term averages in October of 2010 and continued to warm though the summer months coincident with three cyclones passing in close proximity to coastal WA coral reefs. Simultaneously, a near-record strength Leeuwin current transported a large mass of anomalously warm water southwards into temperate regions through January to April 2011 [36]. Here, for the first time, we document mass bleaching and loss of corals across more than 12° of latitude from tropical to temperate waters along the WA coast.

## Materials and Methods

### SST and Climatological Data

Regional-scale temperature anomalies and heat stress data were obtained from the United States National Oceanic and Atmospheric Administration (NOAA) Coral Reef Watch (CRW) E50 product. Advanced Very High Resolution Radiometer (AVHRR) SST data from sensors onboard Polar-orbiting Observational Environmental Satellites (POES) at 0.5° (50 km ×50 km) resolution [37] provided a broad indication of regional temperature profiles which guided selection of satellite-derived metrics for subsequent analyses. The Degree Heating Week (DHW) temperature stress metric used for this product represents the moving cumulative 12-week thermal stress index accumulated once SSTs exceed the maximum monthly mean temperature for that pixel taken from long-term NOAA climatologies [38]. Across regions, *in situ* temperature loggers (Sensus Reefnet Ultra) were deployed at 2–4 m at representative locations to corroborate satellite-derived temperature metrics. Tropical storm and cyclone track data were obtained from the Australian Bureau of Meteorology (BoM).

Satellite temperature metrics used for statistical models however were obtained from *ReefTemp*, a satellite mosaic product of the Commonwealth Scientific and Industrial Research Organisation (CSIRO) Marine and Atmospheric Research unit (http://www.cmar.csiro.au/remotesensing/reeftemp/web/ReefTemp.htm). We used this product over the NOAA CRW product as it provided us with a much finer-scale spatial resolution (ca. [1.8 km]^2^ versus ca. [50 km]^2^), thus increasing explanatory power and providing better model fit. Raw data for *ReefTemp* is administered through the BoM using data collected with AVHRR/3 sensors onboard NOAA-15 and NOAA-17 POES at 0.18° resolution (1.8×1.8 km) [22]. Daily temperature stress metrics for the *ReefTemp* product are calculated by month against long-term averages from CSIRO’s 1993–2003 satellite SST climatology for Australian waters [22]. From this climatology, daily SST anomalies (SSTAs) were calculated as the number of degrees above the long-term average temperature observed for each month. Cumulative temperature stress measured in Degree Heating Days (maxDHD) are therefore calculated as the sum of positive deviations over the climatological monthly means accumulated over the 3 month operational period of the product from the beginning of December to the end February during the Austral summer. We subsequently used this metric in our statistical models as cumulative temperature stress is generally considered to be the most informative variable in predicting coral bleaching [18], [20]. Where pixels consistently returned ‘no data’ values from edge effects caused by proximity to land, data from the next-nearest seaward pixel with a continuous record was used.

As the relative ratio between sun and shade, i.e. irradiance and cloud cover, can also influence the extent and intensity of coral bleaching [23], [25], we used BoM’s daily solar exposure product as a reliable proxy for both insolation, and inversely cloudiness and shading, at the coral surface [24]. Satellite-derived solar exposure estimates in MJ/m^−2^ (megajoules per metre squared) were obtained for the nearest BoM station to survey sites using data captured by MTSAT-1R, MTSAT-2 satellites and Geostationary Meteorological Satellite-5, Geostationary Operational Environmental Satellite-9 from the Japan Meteorological Agency and NOAA [39]. As data from this product was available for land-based stations only, we fitted a spatial spline to extrapolate this data to survey sites, which in most instances were <20 km from the nearest land station.

### Survey Locations

**Figure 1:**
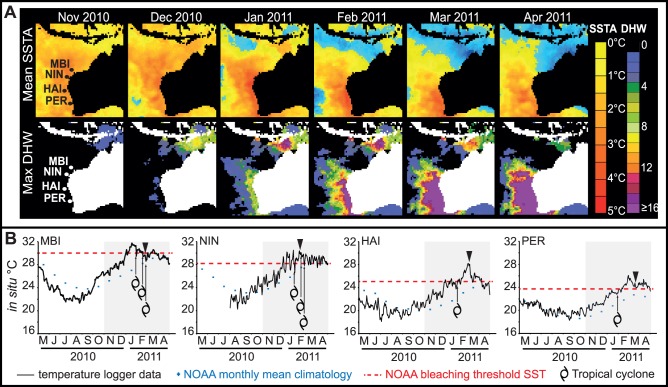
Satellite SST anomalies, thermal stress and *in situ* temperature profiles. (**A**) Onset and evolution of NOAA Coral Reef Watch Sea Surface Temperature Anomalies (SSTA) and Degree Heating Week (DHW) metrics at 0.5°-latitude resolution during the 6 month period over the summer of 2010–11 overlaid with location of surveyed regions. (**B**) Indicative regional *in-situ* temperature logger records. (▾) indicate timing of coral bleaching surveys. (Abbreviations: **MBI** – Montebello and Barrow Islands; **NIN** – Ningaloo Reef; **HAI** – Houtman Abrolhos Islands; and, **PER** – Perth metropolitan waters.).

Surveys of coral cover and bleaching were conducted in the areas of dominant coral reef habitat in WA along more than 1200 km of coastline between the Montebello and Barrow Islands (MBI) in the tropical north (20° 27′S) and Perth (PER) in the temperate south (32° 1′S), (Figure 1a). All surveys were carried out in accordance with the conditions of permits issued by the WA Department of Environment and Conservation, the WA Department of Fisheries and the Rottnest Island Authority. Surveyed locations form part of ongoing long-term benthic monitoring programs of our respective institutions, with permanently marked fixed transects in most locations. As multiple institutions were involved in data collection for this study, sampling and subsequent analyses were divided in two parts: firstly, an assessment was made of the extent and intensity of coral bleaching across study sites; and, secondly, overall change in coral cover before and after the bleaching event was assessed as a proxy for mortality from disturbance. In total, extent of coral bleaching was surveyed along 93 transects (3599 m^2^), and coral cover change along 119 transects (4487 m^2^) across regions with each region divided into distinct geographic locations (Table 1). All surveys were conducted in less than 10 m of water at locations sheltered, or partially sheltered, from prevailing wave energy.

**Table 1:** Breakdown of sampling design, replication of transects and area surveyed for quantitative bleaching and coral change surveys.

			*Bleaching transects*		*Coral change transects*		
Region	Location	Depth (m)	# (area)	Date	# (area)	Pre	Post
*Montebello*/*Barrow*	North	1–8	8 (327 m^2^)	Feb-11	16 (656 m^2^)	Apr-10	Jun-11
*Islands* (MBI)	Lowendal Shelf	1–8	20 (815 m^2^)	”	20 (810 m^2^)	”	”
	Barrow Island	3–5	8 (480 m^2^)	”	12 (486 m^2^)	”	”
	Barrow Shoals	2–3	8 (482 m^2^)	”	8 (326 m^2^)	”	”
*Ningaloo Reef*	Gulf	3–5	5 (78 m^2^)	Feb-11	12 (480 m^2^)	Jan-10	Aug-11
(NIN)	North	1–3	8 (114 m^2^)	”	18 (717 m^2^)	”	”
	Coral Bay	1–4	6 (75 m^2^)	”	12 (459 m^2^)	”	”
	South	1–2	6 (76 m^2^)	”	9 (361 m^2^)	”	”
*Houtman Abrolhos*	Abrolhos	6–9	12 (960 m^2^)	Feb-11/	–	–	–
*Islands* (HAI)				May-11			
*Perth Metropolitan*	Perth	2–9	12 (192 m^2^)	Mar-11	12 (192 m^2^)	Mar-09*	Oct-11
(PER)							
		**TOTAL**	**93 (3599** **m^2^)**		**119 (4487** **m^2^)**		

* with the exception of a subset of transects for which no data was available prior to bleaching (see methods for full description).

### Sampling Design and Data Collection

The data presented in this study were compiled from multiple research and monitoring programs across agencies, and collected with subtly differing methodologies. In most instances, sites were sampled three times: once at least one year prior to bleaching, once during or just after the temperature maxima, and once after the bleaching event when negligible paling of colonies was evident. The exceptions were a subset of transects in PER for which no coral cover data was available prior to bleaching and the Houtman Abrolhos Islands (HAI), where appreciable bleaching was evident during follow-up surveys. As surveys along some transects in PER occurred very near to the temperature maximum, it was assumed that no coral was yet lost and thus total cover of all coral observed (bleached and unbleached) during this survey was a reasonable proxy for total cover before the warming. At the HAI, bleaching data were averaged between March and May 2011 surveys as the bleaching episode was still evolving at the time of the March survey. Consequently, averaged data from the HAI were included in the bleaching assessment, however coral change data were excluded from analyses as it is possible that further change in cover occurred after our final survey. As resolution of imagery was not always sufficient to permit collection of quantitative information on assemblage composition due to turbidity and low light in some locations, we compiled a qualitative list of the dominant coral families and an indication of their relative abundance using visual assessments from researchers that conducted the in-water surveys.

The timing of the bleaching surveys generally corresponded well with the peak in temperature between late January and early March 2011 (Figure 1b). Specifically, MBI was surveyed between February 11–18; NIN between January 25–Feb 18; HAI between 22 February –5 March and PER on 14 March. Coral bleaching was assessed along existing fixed transects with the exception of NIN and PER, where bleaching assessments were made along transects in similar habitat proximal to fixed sites used to assess temporal change in coral cover. Coral cover change was surveyed along existing long-term monitoring sites across regions except PER, where random surveys were conducted. Additionally, for areas not quantitatively surveyed, semi-quantitative observations of both coral cover and coral bleaching were obtained from managers, scientists and industry working in coral reef areas between Port Hedland and Jurien Bay. Observations were standardized to include only estimates of percent cover, and included only instances where bleaching was clearly evident.

Benthic surveys were conducted along six to twenty transects of 20–100 m length per location using either digital photos or stereo video. Subsequent image analyses collected bleaching and coral cover data as percent of cover using coral point count methods from digital still images or by overlaying a grid over selected frames of video footage, both commonly accepted and widely used methods for assessing benthic cover and disturbance [40], [41]. Nearest distance to storm track data was obtained by spatial analysis of Euclidean distances in ArcGIS 10.1 and represented the minimum distance from survey locations to any of the cyclones that formed during the 2010–11 summer. As a minimum, five random or fixed points were surveyed per image, with between 32 and 80 images sampled per transect. To verify the compatibility of analysis techniques a single trained observer independently assessed the two methods of analysing imagery and found no significant differences for percent cover (Kruskal-Wallis *H*_2,48_ = 2.377, *p* = 0.305 ), bleaching (Kruskal-Wallis *H*_2,48_ = 1.411, *p* = 0.494) or the precision of those estimates (Kruskal-Wallis_(*coral cover*)_ *H*_2,12_ = 0.500, *p* = 0.779, Kruskal-Wallis_(*bleaching*)_ *H*_2,12_ = 0.500, *p* = 0.789).

For the bleaching assessments, partial and complete bleaching were combined as any visible loss of pigment indicating a significant loss of zooxanthellae consequently represented a considerable stress to corals [9], [42]. We were conservative in our classification of bleaching, and recorded only those instances where corals appeared visibly paler than photos of the same coral from earlier years in the same season when we were certain that the bleaching threat was absent.

### Data Analyses

Coral bleaching and coral cover change were analysed using General Additive Mixed Models (GAMM) [43] in R v.15.1. Model selection based on multi-model inference was calculated with the ‘MuMIn’ package and resulting model statistics obtained using the ‘mgcv’ package. To avoid over-fitting the GAMM, smoothers were restricted to three or less. For the coral bleaching analysis the dependent variable was proportion of coral bleached and independent variables were location, maxDHD, solar exposure, depth, latitude and longitude. For the coral cover change analysis the dependent variable was the absolute change in coral cover from pre-bleaching levels and the independent variables were location, proportion of bleaching, depth, nearest distance to storm track, pre-bleaching coral cover, latitude and longitude. In both analyses the explanatory variables were chosen on the basis that we had spatially relevant estimates of each and previous studies indicate they can influence the extent of coral bleaching and mortality [8], [18], [23], [44]. The random effect in both analyses were transects within location. For both coral bleaching and coral cover change analyses, final model selection was determined by calculating the second-order Akaike Information Criterion (AICc) [45] for all potential combinations of independent variables using the MuMIn package. We considered the model with the lowest AICc and all other models within 2 AICc units. Where multiple models had similar AICc values, criteria for selection of the best model considered the model containing the fewest variables which accounted for the most overall AIC weight (AICw) [45].

## Results

### SST and Cyclones

Profiles from NOAA satellite SST data showed that temperature maxima followed a clear southward path from tropical to temperate regions with the MBI attaining maximum temperatures in early-mid January, followed by NIN in mid-January, the HAI at the end of February and PER during the first week of March (Figure 1a). Deviations above long-term averages were ∼2°C in the northern regions (MBI and NIN), and up to 5°C in the southern regions (HAI and PER) (Figure 1a). The magnitude of thermal stress (DHW) increased, particularly south of NIN (Figure 1a), until at least May 2011 and exceeded thresholds that are normally associated with mass bleaching (DHW ≥ 4°C weeks) and mortality (DHW ≥ 8°C weeks) (Figure 1a). At higher latitudes, HAI and PER accumulated >30 DHWs whilst the magnitude was less acute in the MBI, with 4–8 DHWs observed. *In situ* water temperatures supported the regional patterns in SST anomalies. Temperature logger records confirmed that bleaching thresholds, defined as +1°C above the maximum long-term monthly mean, were exceeded by 0.5–3°C at all locations (Figure 1b). From 1 December 2010 to 30 April 2011 thresholds were exceeded by 47 days at the MBI, 97 days at NIN and 78 days at both HAI and PER (Figure 1b).

**Figure 2:**
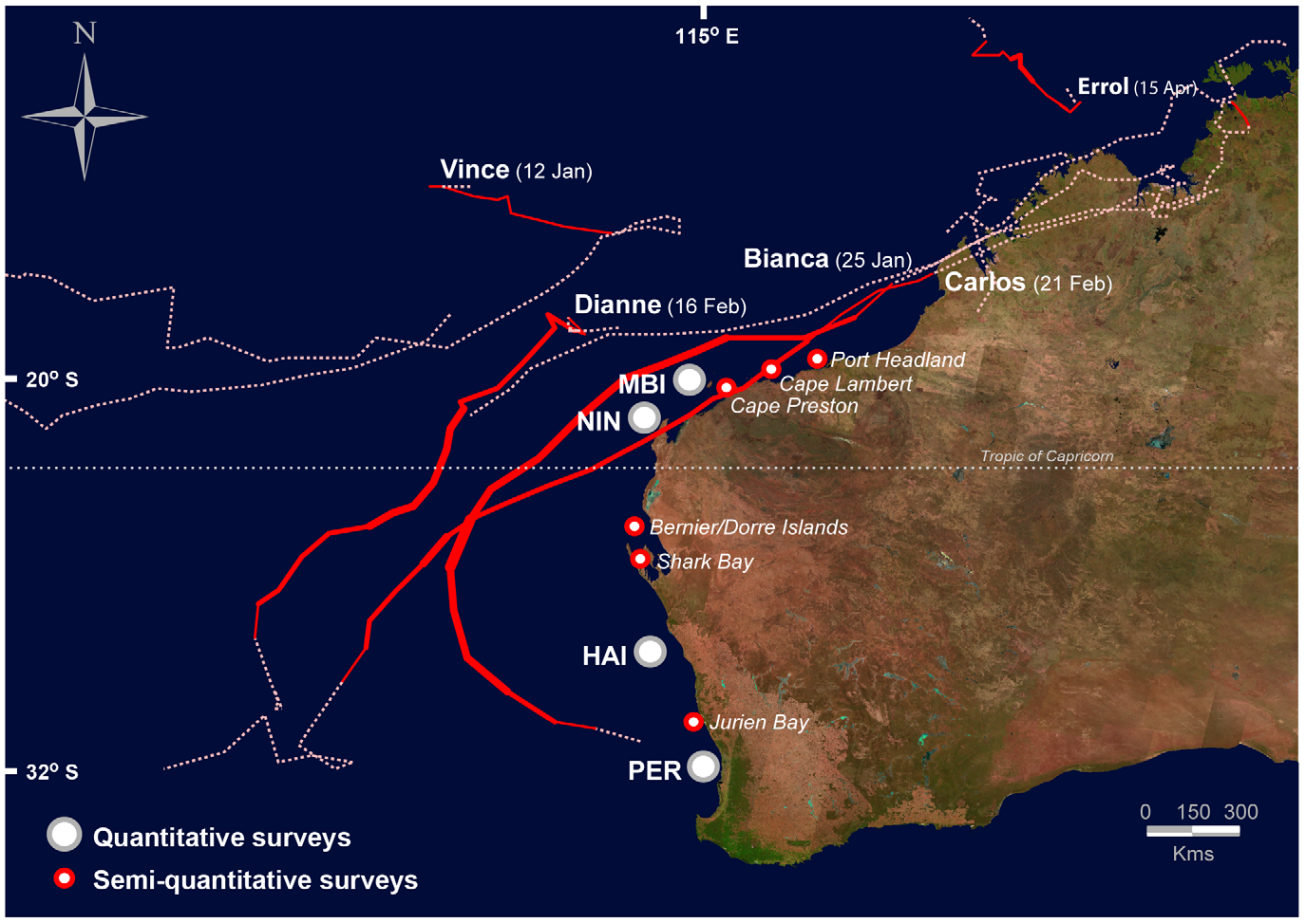
Map of cyclone tracks and survey locations. Map of cyclone tracks for the period November 2010– May 2011. Cyclone tracks shown as solid lines with thickness of the tracks proportional to strength category (maximum strength category 4); dashed-lines indicate tropical low before or after cyclone designation. Locations of quantitative and semi-quantitative survey sites shown as filled circles (see legend in graph).

Considerable storminess and cyclonic activity also occurred during the period of bleaching. Five tropical lows and five cyclones (Bianca, Carlos, Dianne, Errol, Vince) formed within 1000 km of the WA coast (Figure 2), with cyclones Bianca and Carlos passing within 400 km of northern and 700 km of southern sites, at or near the time of maximum heating. Notably, category two cyclone Bianca tracked along the coastline and passed through the MBI and NIN regions and directly over Gulf and North NIN locations (Figure 2). This same storm continued southwards passing within 500 km of the HAI before weakening into a tropical low several hundred kilometres to the north-west of PER.

### Taxonomic Information

Corals from the family Acroporidae were the dominant, or equal most dominant, taxa across three of the four surveyed regions (Table 2). In the MBI, the coral assemblage comprised of roughly equivalent proportions of acroporids, poritids and faviids. NIN and HAI were more similar where acroporids dominated the assemblage, with lesser proportions of faviids and poritids. In contrast, acroporid corals were the least dominant taxa across PER, with pocilloporids and faviids more abundant in relative terms.

**Table 2:** Qualitative community composition of dominant family groups by region. (Numbering is proportional to relative contribution to overall coral assemblage. Higher values denote greater relative contribution.).

	Acroporidae	Poritidae	Faviidae	Pocilloporidae
**Region**				
*MBI*	1	1	1	
*NIN*	3	1	2	
*HAI*	3		2	1
*PER*	1		3	2

### Coral Bleaching Assessment

**Figure 3:**
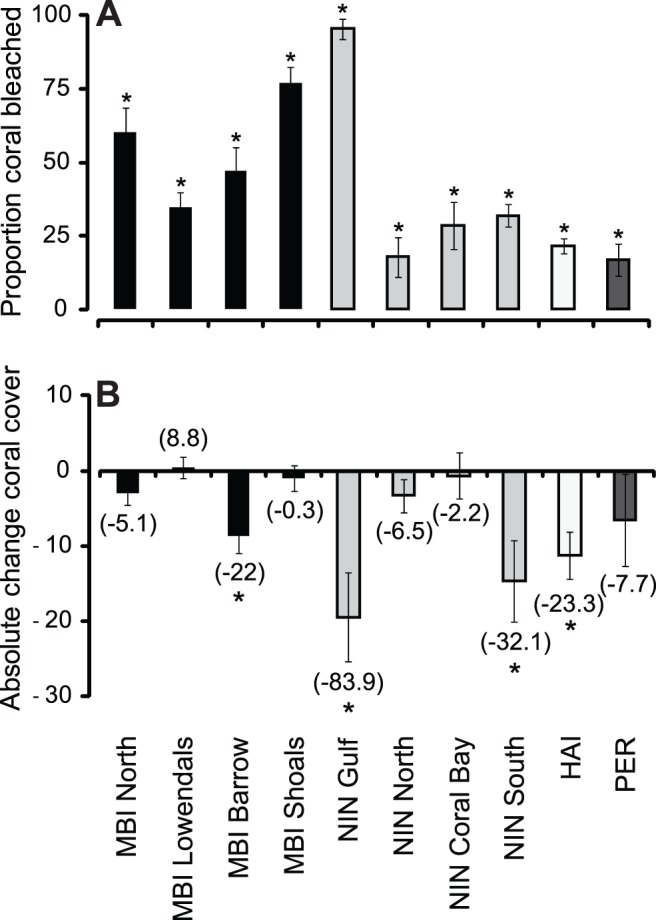
Mean proportion of coral bleaching and absolute change in coral cover. (**A**) Mean proportion of coral bleached by location. (**B**) Mean change in absolute coral cover (values in parentheses represent proportional change in coral cover). Error bars represent ±1SE. (*) denotes significant difference from 0 where 95% confidence intervals failed to intersect the horizontal axis.

Anomalously high water temperatures caused widespread bleaching along much of the WA coastline south of MBI, although the extent of bleaching varied within and among regions. Highest bleaching was observed in the Exmouth Gulf in the NIN region, where 95% (±3.5) [mean (± se)] of all coral bleached. Conversely, at the southern extent of our study, 17% (±5.5) of coral bleached in PER. Regionally, MBI experienced high levels of bleaching across all locations, with ∼77% (±5.3) of corals bleaching on the Barrow Shoals and ∼34% (±5.3) on the Lowendal Shelf (Figure 3a). Significant regional-scale bleaching was also observed at NIN, with high bleaching of reefs in the North (18% ±6.7), Coral Bay (29% ±8.0), and in the South (32% ±3.9) (Figure 3a). HAI bleaching intensity was intermediate between the northernmost and southernmost locations, with 22% (±2.7) bleaching (Figure 3a). For regions and locations not explicitly included in the present study, semi-quantitative observations suggest that considerable bleaching also occurred outside our study areas. From the Pilbara inshore waters at Cape Lambert to corals in Jurien Bay (10° of latitude to the south) 5–100% bleaching was recorded with highest figures recorded for some reefs within Shark Bay (Table 3).

**Table 3:** Semi-quantitative observations of bleaching across WA (Abbreviations: LIT Line intercept transect; PT Photo-transect; V Visual assessment).

Region	Lat.	Method	Coral cover	Bleaching	Date	Source
*Port Hedland*	−20.31	LIT	10–25%	0%	Jan 11	T. Ayling (*Sea Research Pty. Ltd.*)
*Cape Lambert*	−20.59	PT	10–25%	20–40%	Mar 11	J. Stoddart (*MScience*)
*Cape Preston*	−20.85	LIT	30–45%	5–6%	Apr 11	T. Ayling (*Sea Research Pty. Ltd.*)
*Bernier Island*	−24.79	V	0–10%	10–20%	Mar 11	W. Moroney (*DEC*)
*Dorre Island*	−25.11	V	11–30%	20–50%	Mar 11	W. Moroney (*DEC*)
*Shark Bay*	−25.64	LIT	15–45%	30–100%	Apr 11	D. Holley (*DEC*)
*Jurien Bay*	−30.33	LIT	13–35%	8–54%	Mar 11	M. Rule (*DEC*)

**Figure 4:**
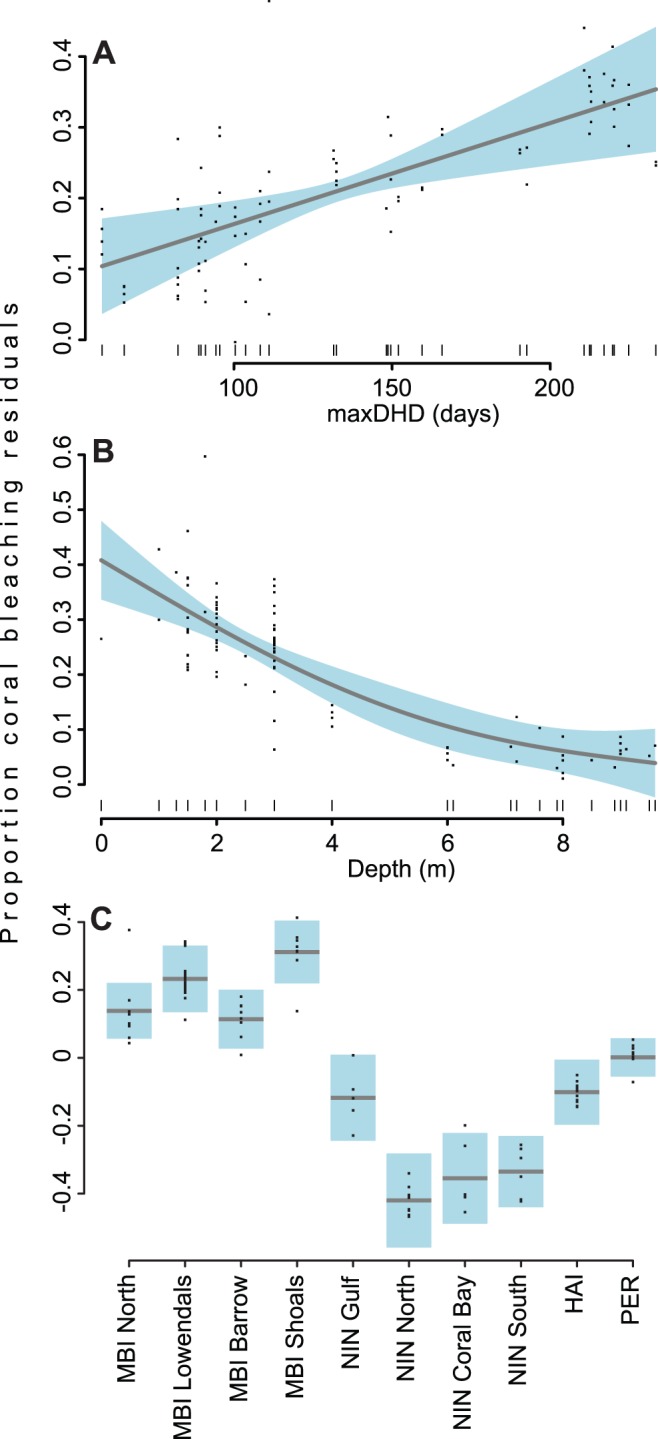
Partial residual plots of best GAM model predictor variables for coral bleaching analysis. Partial residual plots of the modeled relationships between proportion of bleaching (y-axes), (**A**) maxDHD, (**B**) depth, and (**C**) location (mean-centered). Grey lines represent model-fitted splines of the estimated smoothing functions bounded by 95% confidence limits (solid blue shading). Data points represent distribution of raw partial residuals.

The best overall model for explaining observed bleaching patterns accounted for 75% of overall variability and contained significant location, maxDHD and depth parameters (Table 4). A positive relationship existed between proportion of coral bleached and maxDHD (GAMM *F*_1.402_ = 5.549, *p*<0.05) indicating that proportion of bleaching was a strong function of increasing temperature accumulation over the three month summer period (Figure 4a). An inverse relationship was detected between depth and bleaching (GAMM *F*_1_ = 62.686, *p*<1×10^−10^), driven by higher incidence of bleaching on shallow reefs (<4 m) and lower than expected bleaching on deeper reefs (4–10 m) (Figure 4b). Similarly, there was significant spatial heterogeneity in observed proportion of bleaching (GAMM *F*_9_ = 10.46, *p*<1×10^−9^), with higher than expected bleaching in the MBI, less than expected at NIN and the HAI, with the exception of Gulf reefs, and expected levels for PER (Figure 4c).

**Table 4:** Best-subset GAM models for predicting coral bleaching and coral cover change across regions and locations.

MODEL TERMS	*Adj. R^2^*	*AICc*	*AICw*
*Coral bleaching analysis*			
Location^***^, Depth^***^, maxDHD^*^	**0.75**	**213.1**	**0.600**
*Coral cover change analysis*			
Location***, Depth*, Nearest distance**, Pre-bleach cover***	0.37	873.2	0.540
Location***, Nearest distance, Pre-bleach cover***	**0.33**	**875.0**	**0.213**

Adj. R^2^: adjusted coefficient of determination; AICc: corrected Akaike’s Information Criterion; AICw: AIC weight. The best models presented are highlighted in **bold**; alternative models within 2 AICc units are also presented for comparison. (*<0.05, **<0.005, ***<0.0005).

### Coral Cover Change Assessment

Across regions, coral cover change was variable, with significant loss of coral at four of ten locations (Figure 3b). Coral cover was generally stable across the MBI region, with the exception of reefs around Barrow Island which lost a significant −8.6% (±2.3) [−22%] {mean (± se) [proportional % change])} of cover. The greatest amount of coral loss was detected across NIN, with significant losses recorded for the Gulf and the South, with average losses of −19.5% (±5.9) [−83.9%] and −14.7% (±5.4) [−32.1%] respectively (Figure 3b). In the HAI, significant declines in coral cover were observed with an average −11.3% (±6.9) [−23.3%] decrease in cover (Figure 3b).

**Figure 5:**
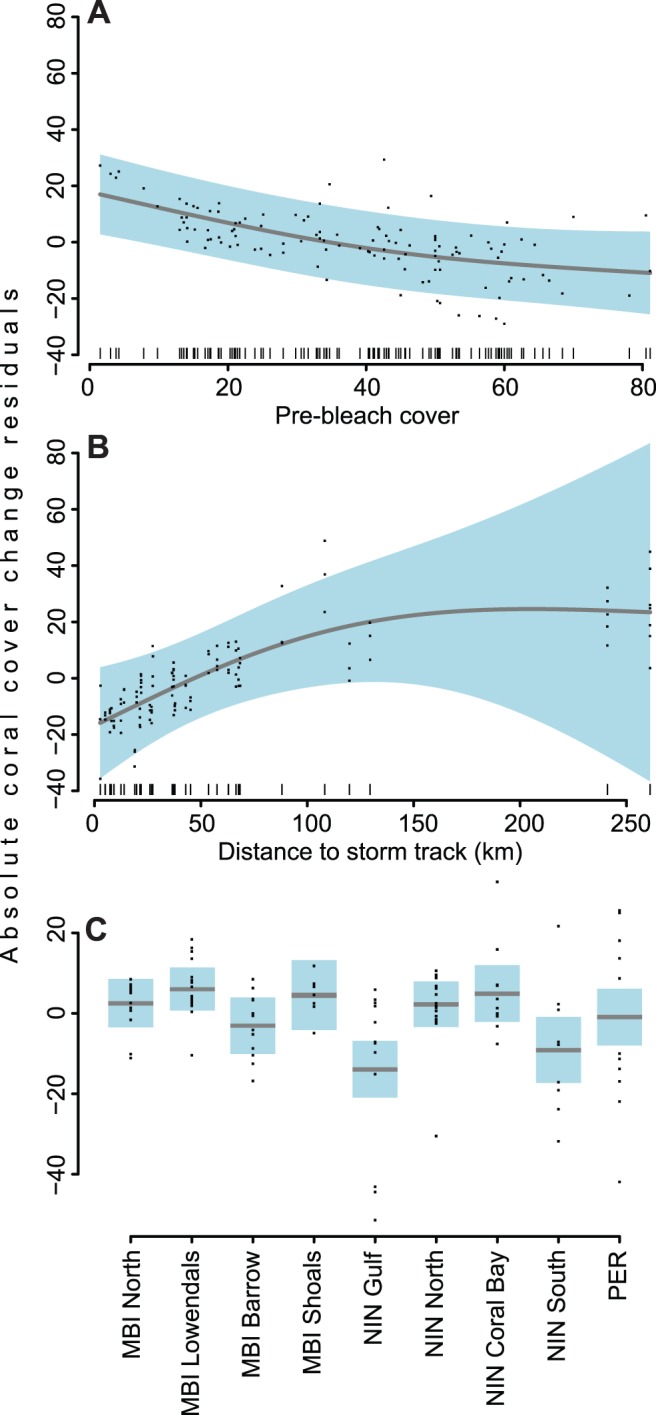
Partial residual plots of best GAM model predictor variables for coral cover change analysis. Partial residual plots of the modeled relationship between absolute change in coral cover (y-axes), (**A**) pre-bleaching coral cover, (**B**) minimum distance from cyclone track, and (**C**) location (mean-centered). Grey lines indicates model-fitted splines of the estimated smoothing function bounded by 95% confidence limits (solid blue shading). Data points represent distribution of raw partial residuals.

The best model for assessing coral cover change included location, nearest distance to storm track and pre-bleaching coral cover variables and explained 33% of variation. A significant inverse relationship between change in coral cover and pre-bleaching coral cover was found (GAMM *F*_1.72_ = 24.636, *p*<0.0001) (Figure 5a). Summed AIC weights indicated that nearest distance to storm track was also an important contributing variable in determining coral change, although not a significant term on its own due to high variance around model estimates especially with increasing distance from storm track (Figure 5b). We also found significant spatial heterogeneity in coral cover change (GAMM *F*_8_ = 9.414, *p*<0.0001) with the model generally explaining coral loss well across locations, with the exception of Ningaloo Gulf and South which lost a greater than expected amount of coral cover (Figure 5c). Although several of the best subset models fell within 2 AICc units, model selection favours parsimony hence the best and simplest three term model which included location, nearest distance to storm track and pre-bleaching coral cover was chosen (Table 4).

## Discussion

Strong La-Niña conditions drove anomalously high water temperatures and storminess along the WA coast during the summer of 2010–11. Across more than 12° of latitude from tropical to temperate waters, significant and widespread coral bleaching followed by spatially patchy loss of coral were recorded. Whilst bleaching levels and post-disturbance loss of coral cover recorded here are comparable to similar large-scale events in the Caribbean [8], [27] and Great Barrier Reef [5], given the extent and intensity of thermal stress and storminess, resultant impacts are less than might be expected given the magnitude of the anomalies observed here [3], [8], [9]. However, we cannot discount continued stress to corals from disease or predation, which often occur when the coral immune response is compromised by disturbance such as bleaching and breakage [31], [33].

Rising SSTs have previously been identified as the main threat to coral reefs along the NW coast of Australia, however, these threats are considered to be relatively low in comparison to other coral reef regions around the world [46], [47]. Regionally, strong El-Niño conditions in 1998 resulted in unprecedented global-scale coral bleaching, with substantial impacts across Indian Ocean coral reefs [4], [48], [49]. Whilst considerable bleaching and mortality of corals on oceanic reefs and atolls in offshore waters of WA were associated with this event [35], there is no evidence to suggest that abnormally high SSTs had resulted in mass bleaching over the extensive area studied here. Indeed, coral bleaching at large spatial scales was previously considered unlikely given the presence of cooling northward-flowing counter-currents running directly adjacent to the WA coastline [50], [51]. Our results, however, clearly demonstrate that cooling counter-currents are not always present or strong enough to mediate the effects of strong southerly flowing warm water currents on corals.

Significantly, we report substantial mass bleaching and loss of corals from predominantly coastal locations across 12° of latitude from tropical to temperate waters. This occurred during one of the strongest recorded La-Niña years when the strength of the Leeuwin current was extremely high, driving exceptionally warm water southward into temperate waters [36]. In comparison, mass coral bleaching episodes reported elsewhere are generally restricted to tropical and subtropical waters [17], [27], [52], [53] and are typically associated with El-Niño events [37]. The implications of our findings, which includes bleaching of corals at the southern limit of reef growth [54], [55], suggests that proposed mechanisms of climate change adaptation, including southward range expansion and thermal refugia [56], may be insufficient in WA. Further, we now have evidence that WA coral communities are susceptible to bleaching under both El-Niño conditions on the offshore atolls [34], [35], and La-Niña conditions in the coastal and near-shore locations in this study. This has important implications for the long-term resilience of corals across the state.

Across regions, location, depth and maxDHD accounted for a large amount of overall variability in observed bleaching. Whilst coral bleaching often results from the interaction between various biological and physical parameters (reviewed by [57]), maxDHD was the best predictor of bleaching during this event and compares well with similar studies assessing environmental drivers of bleaching on broad scales elsewhere [18], [20], [58]. Although temperature was a strong predictor and hence driver of bleaching, this parameter alone cannot account for the variable response of coral communities across locations. Given the spatial scale of the stressor (i.e. size and magnitude of the warm water body), a strong location effect suggests that observed bleaching patterns were mediated by localised factors mitigating the region-wide temperature stress. This is not an uncommon attribute of mass bleaching events, where regional patterns often do not simply downscale to the local sub-regional or reef scale [25], [27], [59].

Whilst bleaching was recorded across all regions and locations, there were distinct patterns in the extent and intensity of the bleaching event. The presence of a strong depth effect indicated that irrespective of region and heating, corals in shallow water (<4 m deep) were more likely to bleach than those in deeper water. The relationship between depth and coral bleaching may partially relate to depth-mediated flow regimes [58], [60], and water column light attenuation properties which reduce incident light reaching the coral surface [61]. Although our study showed that bleaching was more prevalent in shallow water, deep water surveys (15–30 m) around Perth and the Houtman Abrolhos islands revealed considerable bleaching [62], [63], emphasising both the spatial extent and vertical distribution of the 2010/11 warm water event along the WA coast.

Taxonomic composition can also influence observed patterns of bleaching [26]. In this instance, coral communities were relatively similar across much of the study area, however fine-scale differences in coral assemblages and their differential susceptibility to bleaching [19], [64] may also have contributed to observed spatial patterns. This may in part explain bleaching responses for temperate coral communities of Perth, where comparatively resilient faviids constitute a large proportion of the assemblage [55].

The summer of 2010–11 was also very active cyclonically, with multiple storms tracking within close proximity to survey locations at or near the time of heating maxima. Storms and cyclones are likely to influence both bleaching and mortality in complex ways. Our results support other studies which show that tropical storms are generally documented to have a direct and detrimental effect on coral cover when passing within 50–70 km of coral reefs [44], [65]. However they may also alleviate the extent and intensity of bleaching through either lowering temperatures through wind-driven vertical mixing [66], shading reducing incident light intensity thereby mitigating combined heat/light stress [23], [25] or both. Whilst *in-situ* temperature logger records suggest that storms provided no local-scale relief from high temperatures, the weak relationship between bleaching and solar exposure (as a proxy for irradiance after [24]) may have been driven by shading from the associated cloud cover [25]. This highlights the need to consider all potential threatening processes to adequately monitor, describe and understand large-scale disturbance events on coral reefs.

Impacts were observed across a range of taxa including mass fish kills, localised loss of abalone stock and high lobster mortality (reviewed in [36]). Given the severe consequences for other taxa, the thermal profiles for regions in this study and levels of storminess and cyclonic activity, higher mortality of corals might be expected. Although we detected significant and high levels of bleaching across latitudes, resultant declines in coral cover overall were less than expected given known relationships between temperature and coral mortality [58], [67]. Certainly prior coral cover was an important variable in explaining the amount of coral lost, and also not unexpected given that amount of coral that may potentially be lost is a direct function of the amount of coral present. It is possible that we did not detect the full extent of coral loss at some locations, especially for the HAI, however across the remaining regions, timing of post-bleaching surveys was sufficient for any mortality resulting directly from bleaching to be apparent [68], [69]. This may in part be shaped by differential responses to thermal stress displayed by coral communities across regions as a consequence of their prior environmental history. For instance, declining calcification rates for some corals of the Exmouth Gulf are suggestive of a history of chronic disturbance, most likely driven by temperature [70]. Similarly, differences in the distribution of *Symbiodinium* communities in WA [71] may contribute to the spatially heterogeneous patterns in bleaching and coral loss observed here [72], [73]. Further work is urgently needed on threats, and the scale at which those threats operate, in order to better understand the response of, and resultant impacts on, coral communities across WA.

Across the world, coral reefs are increasingly imperiled [13], [14], [74], [75]. Each year there is a steady increase in the number of coral reefs globally reaching the upper limits of their ability to withstand continued disturbance [1]. Regionally, coral reefs of WA are yet to experience cyclic episodes of severe disturbance events seen elsewhere [5], [74], [76]. The patchy response of coral loss observed here under extreme bleaching conditions and widespread storminess suggests resilience amongst WA coral communities. Although storm-driven mortality was most evident in this study, it is also clear that higher latitude corals at the tropical-temperate interface in WA [77] are as susceptible to bleaching as other high-latitude communities elsewhere around Australia [78], [79]. This has important implications under future warming scenarios [80], [81], and questions whether temperate locations will provide appropriate refugia for the long-term survival of coral communities as background warming continues to rise [56].

This study is the first to document disturbance on a regional-scale for Western Australian coral reefs and as such provides a valuable baseline against which to measure the impacts of future disturbance events. Given the spatial extent of coral distribution along WA’s extensive coastline, institutional monitoring programs employing standardised methodologies are crucial in order to understand and document regional-scale disturbance events. This is critical as we continue to develop the ongoing capability to robustly assess the response of WA coral communities to disturbance under scenarios predicting increasing frequency of disturbance cycles [9], [74], [82]. The findings from this study clearly show that regionally, coral communities are susceptible to warming irrespective of their latitudinal situation. However, it is likely that localised biological and environmental parameters interact to mediate bleaching extent and subsequent coral loss at local scales. This highlights the urgent need to incorporate measures of threatening processes into monitoring programs in order to better disentangle the relative importance of different disturbance agents and their subsequent impacts on coral communities [83]. This knowledge is critical in a rapidly changing world where effective and timely adaptive management actions are crucial in order to maximise the resilience of coral communities to global change.
